# Efficacy and safety of esaxerenone (CS-3150) in Japanese patients with type 2 diabetes and macroalbuminuria: a multicenter, single-arm, open-label phase III study

**DOI:** 10.1007/s10157-021-02075-y

**Published:** 2021-06-10

**Authors:** Sadayoshi Ito, Naoki Kashihara, Kenichi Shikata, Masaomi Nangaku, Takashi Wada, Yasuyuki Okuda, Tomoko Sawanobori

**Affiliations:** 1 Division of Nephrology, Endocrinology and Vascular Medicine, Department of Medicine, Tohoku University School of Medicine, 2-1 Seiryo-machi, Aoba-ku, Sendai, Miyagi 980-8575 Japan; 2 Katta General Hospital, Shiroishi, Japan; 3 Department of Nephrology and Hypertension, Kawasaki Medical School, Kurashiki, Japan; 4 Center for Innovative Clinical Medicine, Okayama University Hospital, Okayama, Japan; 5 Division of Nephrology and Endocrinology, Graduate School of Medicine, The University of Tokyo, Tokyo, Japan; 6 Department of Nephrology and Laboratory Medicine, Kanazawa University, Kanazawa, Japan; 7 Data Intelligence Department, Daiichi Sankyo Co., Ltd., Tokyo, Japan; 8 Clinical Development Department, Daiichi Sankyo Co., Ltd., Tokyo, Japan

**Keywords:** Esaxerenone, Macroalbuminuria, Phase III, Type 2 diabetes, Urinary albumin-to-creatinine ratio

## Abstract

**Background:**

Esaxerenone has potential renoprotective effects and reduces the urinary albumin-to-creatinine ratio (UACR) in patients with diabetic kidney disease and overt nephropathy. We investigated the efficacy and safety of esaxerenone in Japanese patients with type 2 diabetes (T2D) and macroalbuminuria (UACR ≥ 300 mg/g creatinine).

**Methods:**

We conducted a multicenter, single-arm, open-label phase III study in 56 patients with T2D and UACR ≥ 300 mg/g creatinine with estimated glomerular filtration rate (eGFR) ≥ 30 mL/min/1.73 m^2^ and treated with a renin–angiotensin system inhibitor. Patients received esaxerenone for 28 weeks at 1.25 mg/day initially with titration to 2.5 mg/day based on serum potassium (K^+^) monitoring. Efficacy was evaluated as the change in UACR from baseline to week 28. Safety endpoints included adverse events (AEs), incidence of serum K^+^ increase, and change in eGFR from baseline.

**Results:**

UACR decreased by 54.6% (95% CI 46.9%, 61.3%) on average from baseline (544.1 mg/g creatinine) to the end of treatment (246.8 mg/g creatinine); 51.8% of patients showed improvement to early nephropathy. AE incidence was 69.6%. Three patients (5.4%) had serum K^+^ levels ≥ 6.0 mEq/L or ≥ 5.5 mEq/L on two consecutive occasions. Hyperkalemia in two patients was transient and resolved during the treatment period. One patient discontinued following two consecutive serum K^+^ values ≥ 5.5 mEq/L. The maximum change from baseline in eGFR was − 8.3 mL/min/1.73 m^2^ at week 24.

**Conclusions:**

Esaxerenone reduced UACR in Japanese patients with T2D and UACR ≥ 300 mg/g creatinine; more than half experienced a transition from UACR ≥ 300 mg/g creatinine to UACR < 300 mg/g creatinine.

**Clinical trial registration:**

JapicCTI-173696

**Supplementary Information:**

The online version contains supplementary material available at 10.1007/s10157-021-02075-y.

## Introduction

Approximately 40% of patients with type 2 diabetes develop diabetic kidney disease (DKD) [1], a progressive condition that represents the leading cause of end-stage kidney disease (ESKD) [1]. Furthermore, DKD has been shown to be associated with an increased risk of cardiovascular morbidity and mortality and a high economic burden [2]. Patients in the overt nephropathy stage of DKD are at particular risk of progression to dialysis and are less likely to show an improvement in nephropathy or disease progression, indicating the critical need for early treatment initiation in this high-risk population [3, 4].

Approaches to preserve kidney function in patients with type 2 diabetes are typically multifactorial and include lifestyle modifications, optimizing glycemic control, and maintaining blood pressure (BP) in the normotensive range [5]. Renin–angiotensin system (RAS) inhibitors (i.e., angiotensin-converting enzyme inhibitors [ACEis] and angiotensin receptor blockers [ARBs]) are also widely used to preserve kidney function in patients with DKD [5]. However, pharmacotherapeutic options for the treatment of DKD remain limited, and available treatments may have inadequate efficacy [6]. Therefore, further options to treat nephropathy and prevent DKD progression to ESKD are needed [5].

Previous studies have shown that reducing albuminuria can decrease the risk of progression to ESKD, and may also decrease the rate of kidney and cardiovascular events. Moreover, clinical studies in patients with DKD have reported an association between treatment-related reductions in albuminuria and lower rates of kidney-related adverse events [7–9], which in turn translates to a lower cardiovascular risk [10, 11]. A recent meta-analysis reported that a > 30% reduction in albuminuria markedly reduced the risk of developing ESKD in patients with diabetes [12, 13], highlighting the clinical significance of therapeutic agents that can reduce albumin excretion. Clinical data suggest that the addition of the mineralocorticoid receptor blockers, spironolactone and eplerenone, to RAS inhibitors reduced urinary albumin levels [14–16], indicating that this class of agents may be used to prevent the progression of nephropathy and may even lead to remission. In addition, a recently published long-term phase III study of finerenone (FIDELIO-DKD) reported that finerenone was associated with reduced decline of estimated glomerular filtration rate (eGFR) compared with placebo, death from renal causes, and chronic kidney disease progression in patients with chronic kidney disease and type 2 diabetes [17]. Although not a current standard therapy for diabetic kidney disease, mineralocorticoid receptor blockers may have potential for this indication if nephroprotective doses can be achieved without unacceptable increases in serum potassium (K^+^).

Esaxerenone, a non-steroidal mineralocorticoid receptor blocker, has demonstrated renoprotective effects in preclinical and clinical studies [18–21]. In a phase II study where esaxerenone was administered for 12 weeks in patients with type 2 diabetes and microalbuminuria (urinary albumin-to-creatinine ratio [UACR] 45– < 300 mg/g creatinine), the UACR was reduced by approximately 50% in a dose-dependent manner [22]. In addition, a 52-week phase III study demonstrated that the percent change in UACR from baseline to the end of treatment was significantly higher with esaxerenone versus placebo in patients with type 2 diabetes and microalbuminuria treated with RAS inhibitors [23]. Based on these studies, an appropriate dose for the treatment of type 2 diabetes with microalbuminuria in terms of efficacy, safety, and tolerability was determined to be 2.5 mg/day. This open-label, 28-week phase III study investigated the safety of once-daily esaxerenone in Japanese patients with type 2 diabetes and macroalbuminuria (UACR ≥ 300 mg/g creatinine), and evaluated the effects of treatment on the UACR.

## Materials and methods

### Study design

This was a multicenter, single-arm, open-label phase III study (JapicCTI-173696) conducted in Japan between September 2017 and November 2018 (Online Resource 1). The study protocol was approved by the local institutional review board at each participating site. The study was conducted in accordance with the principles outlined in the Declaration of Helsinki and ICH-E6-guideline for Good Clinical Practice (CPMP/ICH/135/95). All patients provided written informed consent prior to enrollment in the study. Eligible patients were enrolled in the study using web-based interactive response technology.

### Patients

Eligible patients were aged ≥ 20 years with a diagnosis of both hypertension and type 2 diabetes. All patients had received prior treatment with a RAS inhibitor for at least 3 months, showed UACR ≥ 300 mg/g creatinine in the first morning urine sample on at least two occasions during the pre-study observational run-in period, and had an eGFR of ≥ 30 mL/min/1.73 m^2^ (eGFR was calculated using the following equation for the Japanese population: eGFR = 194 × serum creatinine^−1.094^ × age^−0.287^ [multiplied by 0.739 for female patients]) [24]. Patients who met any of the following exclusion criteria were not eligible for enrollment: presence of type 1 diabetes, glycated hemoglobin (National Glycohemoglobin Standardization Program criteria) ≥ 8.4%, secondary glucose intolerance (such as exocrine pancreatic disease, endocrine disease, severe infection), glomerulonephritis, lupus nephritis, nephrotic syndrome, active nephritis, non-diabetic kidney disease, secondary or malignant hypertension, sitting systolic BP ≥ 160 or < 120 mmHg and sitting diastolic BP ≥ 100 or < 60 mmHg measured during the run-in period, serum K^+^ level < 3.5 or ≥ 5.1 mEq/L in patients with eGFR ≥ 45 mL/min/1.73 m^2^, and serum K^+^  < 3.5 or ≥ 4.8 mEq/L in patients with eGFR ≥ 30 and < 45 mL/min/1.73 m^2^.

### Treatment

Existing treatment with ACEis or ARBs was continued at a constant dosage throughout the study. After a 4-week run-in period, esaxerenone was initiated at a dosage of 1.25 mg/day to minimize the risk of increasing serum K^+^ levels, and then gradually increased to 2.5 mg/day from week 4 onward based on serum K^+^ level monitoring (see Online Resource 2 for full details). The treatment period spanned 28 weeks and the study concluded with a 4-week follow-up period. The use of potassium binders (i.e., spherical adsorption charcoal preparations and ion exchange resins) was prohibited during the study period.

### Data collection

The study visit schedule is presented in Online Resource 3. UACR was evaluated in the first morning urine samples: 3 mL samples were collected by patients and submitted to the medical institution where they were kept refrigerated until analysis at a central laboratory (LSI Medience Co., Ltd.). Urinary albumin and creatinine concentrations were measured from the submitted samples and calculated using the following formula:

$$\begin{gathered} {\text{UACR (mg/g creatinine)}} =\frac{ {\text{urinary albumin concentration }}(\mu {\text{g}}/{\text{mL)}}} {{\text{urine creatinine concentration}}\,{\text{(mg/dL)} \times 100}} .  \end{gathered}$$

Venous blood samples were collected, processed for serum isolation, and stored at − 20 °C or below before being transferred to a central laboratory for analysis. Vital signs were monitored at each study visit, and all adverse events occurring during the study were recorded. Potential causal relationships between adverse events and the study drug were determined by the investigator.

### Efficacy

Efficacy endpoints were the change in UACR from baseline, proportion of patients showing improvement from overt nephropathy to early nephropathy (defined as UACR < 300 mg/g creatinine at two consecutive time points) and change in BP from baseline. In addition, the proportion of patients with a ≥ 30% reduction in UACR and whose UACR decreased to < 300 mg/g creatinine in two consecutive measurements was calculated as a post hoc analysis.

### Safety

The safety endpoints were adverse events, incidence of serum K^+^ increase (≥ 6.0 mEq/L or ≥ 5.5 mEq/L on two consecutive measurements), and change in eGFR from baseline. As a post hoc analysis, the percentage of patients who discontinued treatment due to a ≥ 30% decrease in eGFR from baseline on two consecutive measurements, which was defined as a discontinuation criterion, was evaluated.

### Statistical analysis

For the safety assessment, 45 patients were required to observe at least one patient with an adverse event occurring with a 5% incidence rate. In addition, assuming a ≥ 40% reduction in UACR from baseline (− 0.5108 for the change in a log-transformed value and 0.90 for its standard deviation), a sample size of 35 was required to observe a statistically significant decrease with a power of ≥ 90%. Therefore, the target sample size was set at 50 patients to account for drop-outs.

The safety analysis set included all enrolled patients who received at least one dose of study medication. The full analysis set was used for the efficacy analyses and included all enrolled patients who received at least one dose of study medication and who had at least one efficacy endpoint measurement during the treatment period. Safety endpoints were summarized in a descriptive manner with appropriate summary measures. For UACR, log-transformed values were used to calculate the geometric mean percent change from baseline and its 95% confidence interval (CI) at each time point. The proportion of patients with an improvement from overt nephropathy to early nephropathy, a decrease of ≥ 30%, ≥ 50%, and ≥ 75% in the UACR, was calculated as a percentage. Missing UACR data were not imputed; instead, patients without UACR values for two consecutive time points were classified as not having improved. The two-sided significance level was 5%. All statistical analyses were performed using SAS (version 9.4, SAS Institute Inc., Cary, NC, USA).

## Results

### Patient characteristics

A total of 56 patients were enrolled in the study. Baseline characteristics of the study population are shown in Table 1. All patients had a history of hypertension and the proportions of patients with eGFR < 45, ≥ 45 and < 60, and ≥ 60 mL/min/1.73 m^2^ were 16.1%, 37.5%, and 46.4%, respectively. The esaxerenone dosage was able to be up-titrated to 2.5 mg/day in 96.4% of patients. Eight patients withdrew from the study (four because of adverse events [including two serious adverse events, one with increased serum creatinine and one with decreased eGFR], one who met the serum K^+^ discontinuation criteria, and three who met the eGFR discontinuation criteria).

**Table 1:** Patient demographic and clinical characteristics at baseline

Characteristics	Esaxerenone 1.25–2.5 mg/day, *n* = 56
Sex, male	42 (75.0)
Age, year	65.7 ± 10.2
Weight, kg	71.6 ± 15.8
Body mass index, kg/m^2^	27.2 ± 4.2
Sitting systolic BP, mmHg	143.3 ± 9.4
Sitting diastolic BP, mmHg	83.5 ± 10.2
UACR, mg/g creatinine	569.6 ± 174.0
Median (range)	545.5 (323.9–966.5)
eGFR, mL/min/1.73 m^2^	62.1 ± 16.5
Serum K^+^, mEq/L	4.31 ± 0.28
HbA1c, %	7.07 ± 0.66
LDL cholesterol, mg/L	107.2 ± 26.1
Duration of hypertension, year	11.3 ± 10.3
Duration of diabetes, year	17.3 ± 9.2
Other complications, *n* (%)	
Diabetic retinopathy^a^	35 (62.5)
Diabetic neuropathy	21 (37.5)
Hyperlipidemia	41 (73.2)
Hyperuricemia	18 (32.1)
Coronary artery disease	7 (12.5)
Heart failure (≤ NYHA class II)	0 (0.0)
Atrial fibrillation	2 (3.6)
Other cardiac disorder	2 (3.6)
Antihypertensive agents	
ARB	51 (91.1)
ACE inhibitor	5 (8.9)
Other antihypertensive agents	
Calcium channel blocker	42 (75.0)
Diuretics	8 (14.3)
Alpha blocker	3 (5.4)
Beta blocker	4 (7.1)
Number of antihypertensive agents	
Monotherapy	14 (25.0)
Double therapy	28 (50.0)
Triple therapy or more	14 (25.0)
Hypoglycemic agent	
DPP-4 inhibitor	34 (60.7)
SGLT2 inhibitor	11 (19.6)
GLP-1 receptor agonist	3 (5.4)

Data are either *n* (%) or mean ± standard deviation, unless otherwise stated

*ACE* angiotensin-converting enzyme, *ARB* angiotensin receptor blocker, *BP* blood pressure, *DPP-4* dipeptidyl peptidase 4, *eGFR* estimated glomerular filtration rate, *GLP-1* glucagon-like peptide-1, *HbA1c* glycated hemoglobin, *K*^+^ potassium, *LDL* low density lipoprotein, *NYHA* New York Heart Association, *SGLT2* sodium–glucose cotransporter 2, *UACR* urinary albumin-to-creatinine ratio

^a^Based on the Davis classification; included patients diagnosed with simple retinopathy, pre-proliferative retinopathy, or proliferative retinopathy

### Efficacy

**Fig. 1:**
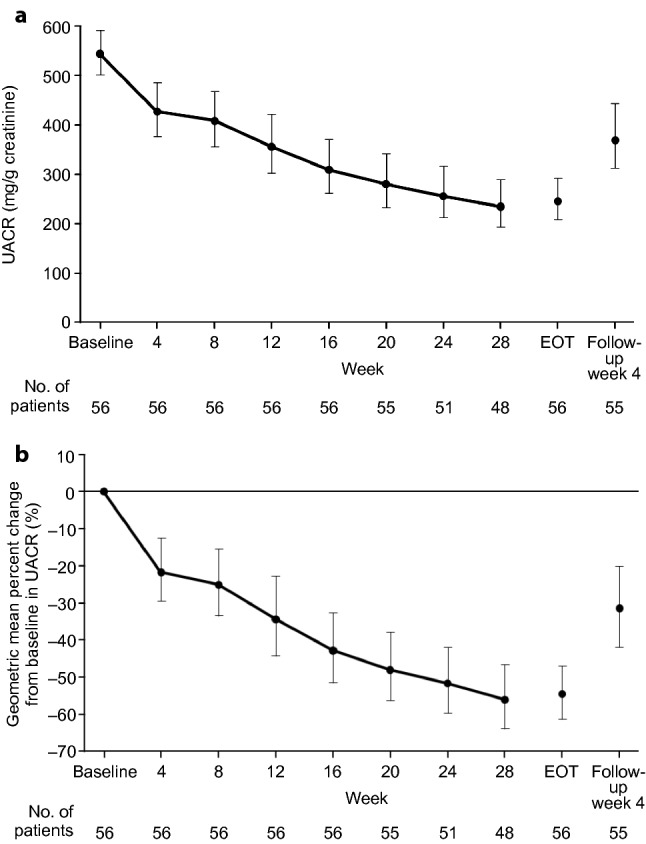
Time course of urinary albumin-to-creatinine ratio (UACR) values (**a**) and geometric mean percent change from baseline in UACR (**b**). End-of-treatment (EOT) values were calculated by taking the average of measurements at the last two visits during the treatment period. Data are shown as geometric mean ± 95% confidence intervals

**Fig. 2:**
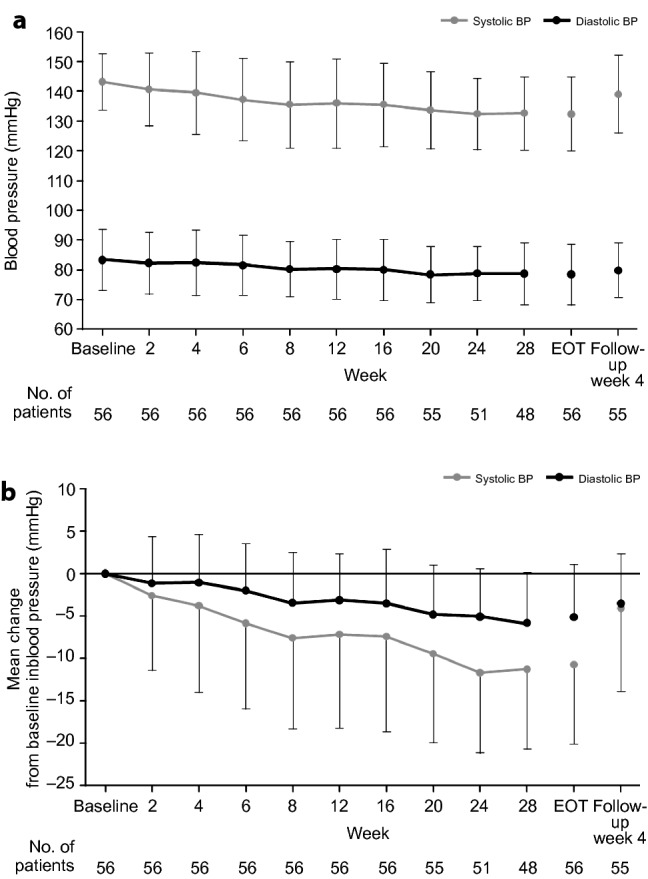
Time course of sitting blood pressure (BP) (**a**) and change in sitting BP from baseline (**b**). Data are mean ± standard deviation. *EOT* end of treatment

At the end of the treatment period, the UACR had statistically significantly decreased from baseline by 54.6% (95% CI 46.9%, 61.3%; from 544.1 mg/g creatinine to 246.8 mg/g creatinine) (Fig. 1). At 4 weeks after the esaxerenone treatment, UACR was 371.2 mg/g creatinine, which was 31.7% lower than at baseline (95% CI 19.9%, 41.8%). Furthermore, subgroup analysis showed that the reduction in UACR occurred in all patient groups, regardless of baseline UACR (< 500 or ≥ 500 mg/g creatinine), eGFR, dipeptidyl peptidase 4 and sodium-glucose cotransporter 2 use, glycated hemoglobin, duration of diabetes, body mass index (< 25 or ≥ 25 kg/m^2^), or number of antihypertensive agents used (Online Resource 4). The proportion of patients who had improved from overt nephropathy to early nephropathy by the end of treatment was 51.8% (95% CI 38.0%, 65.3%). At the end of the treatment period, 75.0%, 57.1%, and 16.1% of patients had ≥ 30%, ≥ 50%, and ≥ 75% reductions from baseline in UACR, respectively (Online Resource 5). The proportion of patients with a ≥ 30% reduction in UACR and whose UACR decreased to < 300 mg/g creatinine was 51.8% at the end of treatment. Systolic BP and diastolic BP decreased gradually during treatment with esaxerenone (systolic BP decreased from 143.3 ± 9.4 mmHg at baseline to 132.6 ± 12.4 mmHg at the end of treatment; diastolic BP decreased from 83.5 ± 10.2 mmHg at baseline to 78.5 ± 10.3 mmHg at the end of treatment), with statistically significant decreases of 10.7 (95% CI 8.2, 13.2) and 5.0 (95% CI 3.4, 6.7) mmHg, respectively, at the end of treatment (Fig. 2).

### Safety

The overall incidence of adverse events was 69.6% (Table 2). The proportion of serious adverse events was 5.4% (*n* = 3; acute myocardial infarction, hypoglycemic unconsciousness, and colon cancer, respectively), but none of these events were considered to have a causal relationship with the study drug. One death occurred (due to myocardial infarction in a patient with multiple pre-existing risk factors); this was also considered to be unrelated to the study drug. The most frequent adverse events occurring in ≥ 5% of patients were viral upper respiratory tract infection, influenza, pharyngitis, hyperuricemia, and contusion (Table 2).

**Table 2:** Summary of treatment-emergent adverse events, adverse events with a frequency of ≥ 5%, and occurrences of increased K^+^

Category of adverse events	Esaxerenone (*n* = 56)
Total number of treatment-emergent adverse events	
Patients with at least one treatment-emergent adverse event	39 (69.6)
Patients with at least one drug-related treatment-emergent adverse event	8 (14.3)
Patients with at least one serious treatment-emergent adverse event	3 (5.4)
Patients with at least one drug-related serious treatment-emergent adverse event	0 (0.0)
Patients who are discontinued from study treatment due to a treatment-emergent adverse event	4 (7.1)
Patients who are discontinued from study treatment due to a drug-related treatment-emergent adverse event	2 (3.6)
Patients who died	1 (1.8)
Frequent adverse events (≥ 5%)	
Viral upper respiratory tract infection	12 (21.4)
Influenza	3 (5.4)
Pharyngitis	3 (5.4)
Hyperuricemia	3 (5.4)
Contusion	3 (5.4)
Increased serum K^+^	
Serum K^+^ ≥ 6.0 mEq/L or ≥ 5.5 mEq/L on two consecutive measurements	3 (5.4)
Discontinuation due to increased serum K^+^	1 (1.8)

Data are *n* (%)

*K*^+^ potassium

The proportion of patients with serum K^+^ levels ≥ 6.0 mEq/L or ≥ 5.5 mEq/L on two consecutive occasions was 5.4% (*n* = 3) (Table 2). Baseline serum K^+^ values in these patients were ≥ 4.5 mEq/L. Two patients with serum K^+^  ≥ 6.0 mEq/L (level 6.1 mEq/L in both) had their esaxerenone dose down-titrated from 2.5 to 1.25 mg/day and were able to complete the 28-week treatment period. One patient receiving esaxerenone 1.25 mg/day had treatment discontinued due to two consecutive serum K^+^ values ≥ 5.5 mEq/L; serum K^+^ levels showed signs of improvement after treatment discontinuation.

**Fig. 3:**
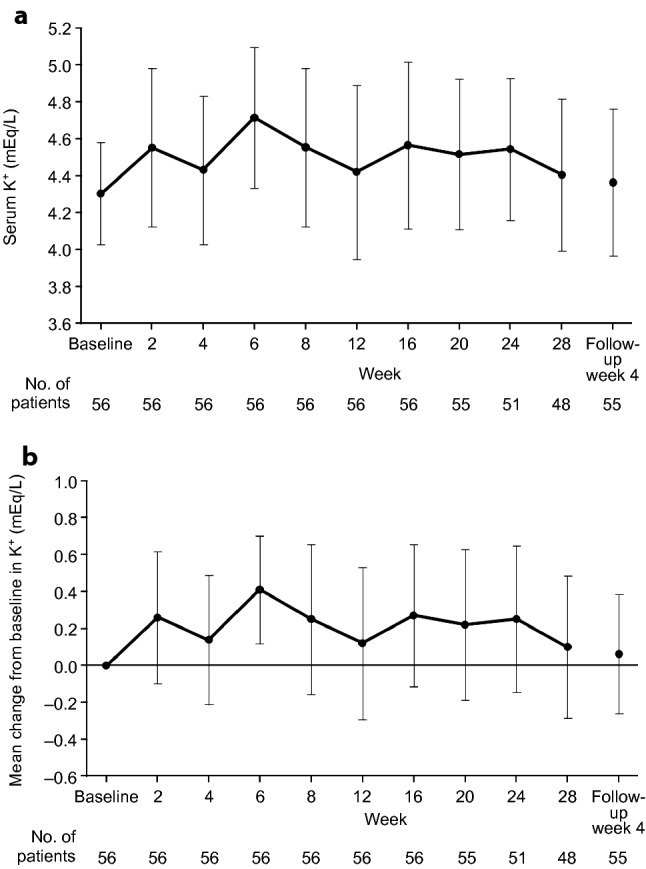
Time course of serum potassium (K^+^) (**a**) and mean change from baseline in serum K^+^ (**b**). Data are mean ± standard deviation

**Fig. 4:**
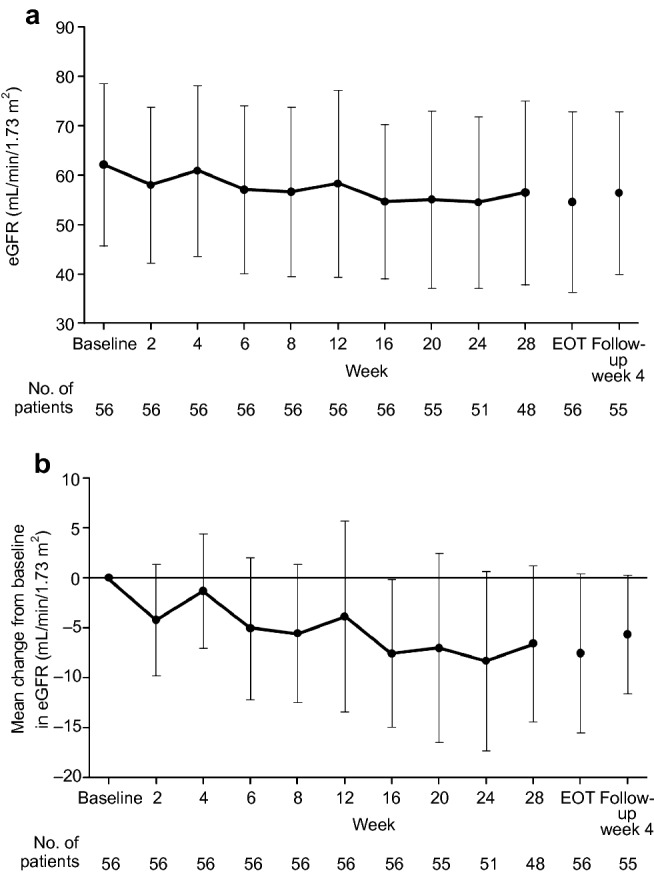
Time course of estimated glomerular filtration rate (eGFR) values (**a**) and mean change from baseline in eGFR (**b**). Data are mean ± standard deviation. *EOT* end of treatment

Episodes of hyperkalemia occurred throughout the treatment period and were not clustered at any specific time point. After esaxerenone treatment initiation, fluctuations in serum K^+^ levels ranged from 0.1 to 0.4 mEq/L, and no further increase in K^+^ level was observed (Fig. 3). eGFR decreased steadily during the study, although reductions appeared to stabilize after week 16 of the treatment period; the mean change from baseline in eGFR was − 8.3 mL/min/1.73 m^2^ at week 24, and levels had returned to near baseline values at 4 weeks after treatment discontinuation (Fig. 4). There were five patients (8.9%) who discontinued treatment due to two consecutive measurements showing a ≥ 30% decrease in eGFR during the treatment period. However, following treatment discontinuation, eGFR levels improved without the need for further intervention.

## Discussion

This study showed that adding esaxerenone to existing RAS inhibitor therapy reduced UACR in patients with type 2 diabetes and UACR ≥ 300 mg/g creatinine, and the majority of patients showed improvement of overt nephropathy to early nephropathy during the 28-week treatment period.

Our previous phase II and III studies have demonstrated the efficacy and safety of esaxerenone in patients with type 2 diabetes and albuminuria (UACR 30– < 1000 mg/g creatinine) or UACR 45– < 300 mg/g creatinine [19, 22, 23]. Improvements in albuminuria observed in the current study are consistent with previous studies and may indicate renoprotection because data from a meta-analysis have shown that a 30% reduction in albuminuria was associated with suppression of subsequent risk of ESKD [12, 25]. A recent study on finerenone, a non-steroidal mineralocorticoid receptor blocker, reported a significant reduction (by about 30%) in UACR as well as a primary composite renal outcome of 17.8% with finerenone versus 21.1% with placebo in patients with type 2 diabetes and albuminuria [17]. In the present study, the reduction in UACR was 54.6% and proportion of patients with a ≥ 30% decrease in UACR was 75.0%. These findings point to a potential event-prevention effect of esaxerenone. Furthermore, even at 4 weeks after the completion of esaxerenone treatment, UACR remained lower than baseline by 31.7%, which was statistically significant. Additional research is needed to evaluate the possibility of improvements in kidney function during long-term therapy with esaxerenone, but the fact that UACR did not immediately rebound to baseline after esaxerenone treatment was stopped in this study might indicate that there were structural improvements in the kidney during esaxerenone therapy. However, 4 weeks of follow-up was not sufficient to determine this change properly.

Five patients (8.9%) discontinued esaxerenone treatment due to decreased eGFR. However, all showed a trend toward recovery of kidney function after completion of treatment. Overall, the reductions in eGFR seen during treatment with esaxerenone were not sustained after treatment was discontinued and eGFR approached baseline levels at 4 weeks after the end of the study, which would be considered acceptable in a clinical setting.

With respect to the hyperkalemia events seen in the present study, 5.4% of patients had increased serum K^+^ levels (≥ 5.5 mEq/L on two consecutive measurements or ≥ 6.0 mEq/L), but increases in serum K^+^ were not associated with any symptoms or electrocardiogram abnormalities. Increased serum K^+^ levels were effectively managed by dose reduction or treatment discontinuation in the present study, and levels showed signs of improvement after therapy was stopped in all patients.

Several limitations need to be taken into account when interpreting current findings, including the open-label study design and lack of a control group, meaning that there is no information on the natural evolution of kidney function in this study population over time. Such information would have been useful to better understand the potential protective effects of esaxerenone on the kidney. Furthermore, the study population included only patients from Japan. The assessment period of 6 months is another limitation of this study, and studies with longer follow-up times are needed. Finally, we did not include patients with overt nephropathy and UACR ≥ 1000 mg/g creatinine in our study; so, the efficacy of esaxerenone in these patients remains to be determined.

## Conclusions

Esaxerenone treatment for 28 weeks reduced UACR in patients with type 2 diabetes and UACR ≥ 300 mg/g creatinine, and more than half of the patients experienced a transition from overt nephropathy to early nephropathy. These findings suggest that esaxerenone might prevent the progression of nephropathy and ultimately ESKD in these patients. Moreover, treatment with esaxerenone increased serum K^+^ levels and decreased eGFR levels. However, the risk of hyperkalemia and a rapid decrease in eGFR could be minimized by initiating esaxerenone treatment at 1.25 mg/day and increasing the dosage to 2.5 mg/day after serum K^+^ levels have been evaluated. Starting esaxerenone treatment for overt nephropathy at low doses and regularly monitoring serum K^+^ and eGFR levels are crucial in ensuring patient safety. In addition, esaxerenone dose reduction or discontinuation should be considered when a patient shows repeated and clinically significant abnormal changes in either or both serum K^+^ and eGFR parameters during treatment.

## Data sharing statement

Deidentified individual participant data and applicable supporting clinical trial documents, which include participant demographics, medical histories, vitals, laboratory test results, adverse events, concomitant medications and final patient statements, may be available on request at https://vivli.org/. Data will be available for sharing after the treatment agent has received marketing approval and after the study results have been accepted for publication. Requests for data will be reviewed by the Independent Review Panel on the basis of the scientific merit of the research proposal and bound by the limitation of participants’ consent. Data requested must execute the Data Use Agreement, and access will be provided for 1 year after the request has been authorized. Requests should be made via the Vivli Platform, through which access to the data will be provided. In cases where clinical study data and supporting documents are provided pursuant to our company policies and procedures, Daiichi Sankyo Co., Ltd. will continue to protect the privacy of our clinical study participants. Access to data may be declined if there is a potential conflict of interest or competitive risk between Daiichi Sankyo Co., Ltd. and the requesting party.

## Supplementary Information

Below is the link to the electronic supplementary material.

Supplementary file1 (PDF 85 kb)

Supplementary file2 (PDF 143 kb)

Supplementary file3 (PDF 109 kb)

Supplementary file4 (docx 27 kb)

Supplementary file5 (PDF 140 kb)

## References

[1] Alicic RZ, Rooney MT, Tuttle KR (2017). Diabetic kidney disease: challenges, progress, and possibilities. Clin J Am Soc Nephrol.

[2] Vupputuri S, Kimes TM, Calloway MO, Christian JB, Bruhn D, Martin AA (2014). The economic burden of progressive chronic kidney disease among patients with type 2 diabetes. J Diabetes Complications.

[3] Babazono T, Nyumura I, Toya K, Hayashi T, Ohta M, Suzuki K (2009). Higher levels of urinary albumin excretion within the normal range predict faster decline in glomerular filtration rate in diabetic patients. Diabetes Care.

[4] Yokoyama H, Araki S, Honjo J, Okizaki S, Yamada D, Shudo R (2013). Association between remission of macroalbuminuria and preservation of renal function in patients with type 2 diabetes with overt proteinuria. Diabetes Care.

[5] Delanaye P, Scheen AJ (2019). Preventing and treating kidney disease in patients with type 2 diabetes. Expert Opin Pharmacother.

[6] Muskiet MHA, Wheeler DC, Heerspink HJL (2019). New pharmacological strategies for protecting kidney function in type 2 diabetes. Lancet Diabetes Endocrinol.

[7] Roscioni SS, Heerspink HJL, de Zeeuw D (2014). Microalbuminuria: target for renoprotective therapy PRO. Kidney Int.

[8] Gaede P, Tarnow L, Vedel P, Parving HH, Pedersen O (2004). Remission to normoalbuminuria during multifactorial treatment preserves kidney function in patients with type 2 diabetes and microalbuminuria. Nephrol Dial Transplant.

[9] Araki S, Haneda M, Koya D, Kashiwagi A, Uzu T, Kikkawa R (2008). Clinical impact of reducing microalbuminuria in patients with type 2 diabetes mellitus. Diabetes Res Clin Pract.

[10] Agewall S, Wikstrand J, Ljungman S, Fagerberg B (1997). Usefulness of microalbuminuria in predicting cardiovascular mortality in treated hypertensive men with and without diabetes mellitus. Risk Factor Intervention Study Group. Am J Cardiol.

[11] Kalaitzidis RG, Bakris GL (2010). Serum creatinine vs. albuminuria as biomarkers for the estimation of cardiovascular risk. Curr Vasc Pharmacol.

[12] Heerspink HJL, Greene T, Tighiouart H, Gansevoort RT, Coresh J, Simon AL (2019). Change in albuminuria as a surrogate endpoint for progression of kidney disease: a meta-analysis of treatment effects in randomised clinical trials. Lancet Diabetes Endocrinol.

[13] Levey AS, Gansevoort RT, Coresh J, Inker LA, Heerspink HL, Grams ME (2020). Change in albuminuria and GFR as end points for clinical trials in early stages of CKD: a scientific workshop sponsored by the National Kidney Foundation in collaboration with the US Food and Drug Administration and European Medicines Agency. Am J Kid Dis.

[14] Mehdi UF, Adams-Huet B, Raskin P, Vega GL, Toto RD (2009). Addition of angiotensin receptor blockade or mineralocorticoid antagonism to maximal angiotensin-converting enzyme inhibition in diabetic nephropathy. J Am Soc Nephrol.

[15] Sato A, Hayashi K, Naruse M, Saruta T (2003). Effectiveness of aldosterone blockade in patients with diabetic nephropathy. Hypertension.

[16] Bomback AS, Kshirsagar AV, Amamoo MA, Klemmer PJ (2008). Change in proteinuria after adding aldosterone blockers to ACE inhibitors or angiotensin receptor blockers in CKD: a systematic review. Am J Kidney Dis.

[17] Bakris GL, Agarwal R, Anker SD, Pitt B, Ruilope LM, Rossing P (2020). Effect of finerenone on chronic kidney disease outcomes in type 2 diabetes. N Engl J Med.

[18] Li L, Guan Y, Kobori H, Morishita A, Kobara H, Masaki T (2019). Effects of the novel nonsteroidal mineralocorticoid receptor blocker, esaxerenone (CS-3150), on blood pressure and urinary angiotensinogen in low-renin Dahl salt-sensitive hypertensive rats. Hypertens Res.

[19] Itoh H, Ito S, Rakugi H, Okuda Y, Nishioka S (2019). Efficacy and safety of dosage-escalation of low-dosage esaxerenone added to a RAS inhibitor in hypertensive patients with type 2 diabetes and albuminuria: a single-arm, open-label study. Hypertens Res.

[20] Arai K, Tsuruoka H, Homma T (2015). CS-3150, a novel non-steroidal mineralocorticoid receptor antagonist, prevents hypertension and cardiorenal injury in Dahl salt-sensitive hypertensive rats. Eur J Pharmacol.

[21] Arai K, Morikawa Y, Ubukata N, Tsuruoka H, Homma T (2016). CS-3150, a novel nonsteroidal mineralocorticoid receptor antagonist, shows preventive and therapeutic effects on renal injury in deoxycorticosterone acetate/salt-induced hypertensive rats. J Pharmacol Exp Ther.

[22] Ito S, Shikata K, Nangaku M, Okuda Y, Sawanobori T (2019). Efficacy and safety of esaxerenone (CS-3150) for the treatment of type 2 diabetes with microalbuminuria: a randomized, double-blind, placebo-controlled, phase II trial. Clin J Am Soc Nephrol.

[23] Ito S, Kashihara N, Shikata K, Nangaku M, Wada T, Okuda Y (2020). Esaxerenone (CS-3150) in patients with type 2 diabetes and microalbuminuria (ESAX-DN): phase 3 randomized controlled clinical trial. Clin J Am Soc Nephrol.

[24] Matsuo S, Imai E, Horio M, Yasuda Y, Tomita K, Nitta K (2009). Revised equations for estimated GFR from serum creatinine in Japan. Am J Kidney Dis.

[25] Coresh J, Heerspink HJL, Sang Y, Matsushita K, Arnlov J, Astor BC (2019). Change in albuminuria and subsequent risk of end-stage kidney disease: an individual participant-level consortium meta-analysis of observational studies. Lancet Diabetes Endocrinol.

