# Supplementary material for: Efficacy and safety of esaxerenone (CS-3150) in Japanese patients with type 2 diabetes and macroalbuminuria: a multicenter, single-arm, open-label phase III study
Source: Clin Exp Nephrol. 2021 Jun 10;25(10):1070–8. doi: 10.1007/s10157-021-02075-y (PMC8421271; doi:10.1007/s10157-021-02075-y)
Supplement: Supplementary file 1 — Supplementary file1 (PDF 85 kb) [file 10157_2021_2075_MOESM1_ESM.pdf]

Electronic supplementary material

Efficacy and safety of esaxerenone (CS-3150) in Japanese patients with type 2 diabetes and macroalbuminuria: a multicenter, single-arm, open-label phase III study

Clinical and Experimental Nephrology

Sadayoshi Ito, Naoki Kashihara, Kenichi Shikata, Masaomi Nangaku, Takashi Wada, Yasuyuki Okuda, Tomoko Sawanobori

Corresponding author:

Sadayoshi Ito, MD, PhD

Division of Nephrology, Endocrinology and Vascular Medicine, Department of Medicine, Tohoku University School of Medicine, 2-1 Seiryo-machi, Aoba-ku, Sendai, Miyagi 980-8575, Japan

E-mail: db554@med.tohoku.ac.jp

Online Resource 2

For patients with an estimated glomerular filtration rate (eGFR) ≥45 mL/min/1.73 m^2 during the run-in period, the esaxerenone dosage was maintained at 1.25 mg/day if the most recent serum potassium (K^+) level was ≥5.1 mEq/L, and increased to 2.5 mg/day if the most recent serum K^+ level was <5.1 mEq/L. In patients with eGFR 30 to <45 mL/min/1.73 m^2 during the run-in period, the esaxerenone dosage was maintained at 1.25 mg/day if the most recent serum K^+ level was ≥4.8 mEq/L, and increased to 2.5 mg/day if the most recent serum K^+ was <4.8 mEq/L.

During treatment with esaxerenone 2.5 mg/day, if the most recent serum K^+ was ≥5.5 to <6.0 mEq/L, serum K^+ was re-measured within 3 days. If the second measurement was <5.5 mEq/L, the esaxerenone dosage was maintained at 2.5 mg/day; if the second measurement was 5.5 to <6.0 mEq/L, treatment was interrupted followed by a clinic visit and dose reduction to 1.25 mg/day; and if the second serum K^+ level was ≥6.0 mEq/L, esaxerenone was discontinued. If the most recent serum K^+ level was ≥6.0 mEq/L, serum K^+ was re-measured within 3 days. If the second value was <5.5 mEq/L, treatment was interrupted followed by a clinic visit and dose reduction to 1.25 mg/day, and if the second value was ≥5.5 mEq/L, esaxerenone was discontinued.

During treatment with esaxerenone 1.25 mg/day, if the most recent serum K^+ measurement was ≥5.5–6.0 mEq/L, serum K^+ was re-measured within 3 days. If the second measurement was <5.5 mEq/L, the esaxerenone dosage was maintained at 1.25 mg/day; if the second value was over 5.5 mEq/L, esaxerenone was discontinued.
