# Supplementary material for: Efficacy and safety of esaxerenone (CS-3150) in Japanese patients with type 2 diabetes and macroalbuminuria: a multicenter, single-arm, open-label phase III study
Source: Clin Exp Nephrol. 2021 Jun 10;25(10):1070–8. doi: 10.1007/s10157-021-02075-y (PMC8421271; doi:10.1007/s10157-021-02075-y)
Supplement: Supplementary file 2 — Supplementary file2 (PDF 143 kb) [file 10157_2021_2075_MOESM2_ESM.pdf]

Electronic supplementary material

Efficacy and safety of esaxerenone (CS-3150) in Japanese patients with type 2 diabetes and macroalbuminuria: a multicenter, single-arm, open-label phase III study

Clinical and Experimental Nephrology

Sadayoshi Ito, Naoki Kashihara, Kenichi Shikata, Masaomi Nangaku, Takashi Wada, Yasuyuki Okuda, Tomoko Sawanobori

Corresponding author:

Sadayoshi Ito, MD, PhD

Division of Nephrology, Endocrinology and Vascular Medicine, Department of Medicine, Tohoku University School of Medicine, 2-1 Seiryo-machi, Aoba-ku, Sendai, Miyagi 980-8575, Japan

E-mail: db554@med.tohoku.ac.jp

Online Resource 3 Study schedule of assessments

**Assessments**	**Run-in period**			**Treatment period**													**Follow-up period**
**Timing of visit (weeks)**	**Start**	**2**	**3[a]**	**Start**	**2**	**4**	**6**	**8**	**12**	**16**	**20**	**24**	**28 (EOT)**	**2 weeks after dose increase**	**After visits where serum K^+ ≥5.5 mEq/L**	**Treatment discontinuation**	**4 weeks after EOT**
Urine sampling																	
UACR	X	X	X[a]			X		X	X	X	X	X	X			X	X
Blood sampling																	
Hematology		X				X			X				X			X	
Biochemistry		X				X			X				X			X	
Potassium		X			X	X	X	X	X	X	X	X	X	X	X[b]	X	X
Creatinine		X			X	X	X	X	X	X	X	X	X	X	X[b]	X	X
Vital signs																	
BP, pulse	X	X			X	X	X	X	X	X	X	X	X	X		X	X
Adverse events				X	X	X	X	X	X	X	X	X	X	X	X	X	X

[a]Assessed at week 3 if urinary albumin-to-creatinine ratio criteria were not met at week 2.

[b]Serum potassium and creatinine measurement only.

BP blood pressure; *EOT* end of treatment; *UACR* urinary albumin-to-creatinine ratio.
