# Supplementary material for: Efficacy and safety of esaxerenone (CS-3150) in Japanese patients with type 2 diabetes and macroalbuminuria: a multicenter, single-arm, open-label phase III study
Source: Clin Exp Nephrol. 2021 Jun 10;25(10):1070–8. doi: 10.1007/s10157-021-02075-y (PMC8421271; doi:10.1007/s10157-021-02075-y)
Supplement: Supplementary file 3 — Supplementary file3 (PDF 109 kb) [file 10157_2021_2075_MOESM3_ESM.pdf]

Electronic supplementary material

Efficacy and safety of esaxerenone (CS-3150) in Japanese patients with type 2 diabetes and macroalbuminuria: a multicenter, single-arm, open-label phase III study

Clinical and Experimental Nephrology

Sadayoshi Ito, Naoki Kashihara, Kenichi Shikata, Masaomi Nangaku, Takashi Wada, Yasuyuki Okuda, Tomoko Sawanobori

Corresponding author:

Sadayoshi Ito, MD, PhD

Division of Nephrology, Endocrinology and Vascular Medicine, Department of Medicine, Tohoku University School of Medicine, 2-1 Seiryo-machi, Aoba-ku, Sendai, Miyagi 980-8575, Japan

E-mail: db554@med.tohoku.ac.jp

Online Resource 5 Proportion of patients with UACR reduction at the end of treatment

**Category**	**Esaxerenone** ***n*** **= 56**	**95% CI**
Patients with improvement in albuminuria[a], *n* (%)	29 (51.8)	[38.0, 65.3]
Patients with transition to microalbuminuria[b], *n* (%)	29 (51.8)	[38.0, 65.3]
Patients with ≥30% reduction in UACR, *n* (%)	42 (75.0)	[61.6, 85.6]
Patients with ≥50% reduction in UACR, *n* (%)	32 (57.1)	[43.2, 70.3]
Patients with ≥75% reduction in UACR, *n* (%)	9 (16.1)	[7.6, 28.3]

[a]Improvement in albuminuria was defined as < 300 mg/g creatinine and 30% reduction from baseline on two consecutive measurements at the end of treatment

[b]Microalbuminuria was defined as two consecutive UACR values of <300 mg/g creatinine at the end of treatment.

CI confidence interval; *UACR* urinary albumin-to-creatinine ratio.
