# Supplementary material for: Efficacy and safety of esaxerenone (CS-3150) in Japanese patients with type 2 diabetes and macroalbuminuria: a multicenter, single-arm, open-label phase III study
Source: Clin Exp Nephrol. 2021 Jun 10;25(10):1070–8. doi: 10.1007/s10157-021-02075-y (PMC8421271; doi:10.1007/s10157-021-02075-y)
Supplement: Supplementary file 4 — Supplementary file4 (docx 27 kb) [file 10157_2021_2075_MOESM4_ESM.docx]

# Electronic supplementary material

# Efficacy and safety of esaxerenone (CS-3150) in Japanese patients with type 2 diabetes and macroalbuminuria: a multicenter, single-arm, open-label phase III study

Clinical and Experimental Nephrology

Sadayoshi Ito, Naoki Kashihara, Kenichi Shikata, Masaomi Nangaku, Takashi Wada, Yasuyuki Okuda, Tomoko Sawanobori

**Corresponding author:**

Sadayoshi Ito, MD, PhD

Division of Nephrology, Endocrinology and Vascular Medicine, Department of Medicine, Tohoku University School of Medicine, 2-1 Seiryo-machi, Aoba-ku, Sendai, Miyagi 980-8575, Japan

E-mail: db554@med.tohoku.ac.jp

**Online Resource 4** Subgroup analysis of change in the urinary albumin-to-creatinine ratio (UACR) from baseline to the end of treatment in patient subgroups

| **Subgroups** | **Mean ± SD** | **Median (Range)** | **Geometric mean % change [95% CI]** |
| --- | --- | --- | --- |
| Change from baseline |  | | |
| Baseline UACR, mg/g creatinine |  | | |
| <500 (*n* = 23) | −170.3 ± 105.0 | −200.2 (−300.5, 59.8) | −47.1 [−56.3, −35.8] |
| ≥500 (*n* = 33) | −340.5 ± 247.4 | −388.1 (−786.0, 470.9) | −59.3 [−67.7, −48.6] |
| Baseline eGFR, mL/min/1.73 m^2^ |  | | |
| <60 (*n* = 30) | −266.6 ± 173.5 | −275.6 (−514.5, 195.1) | −53.5 [−61.4, −43.9] |
| ≥60 (*n* = 26) | −275.3 ± 262.3 | −294.3 (−786.0, 470.9) | −55.9 [−66.6, −41.8] |
| Baseline HbA1c, % |  | | |
| <6.9 (*n* = 20) | −299.7 ± 184.9 | −293.1 (−786.0, 51.5) | −55.7 [−65.5, −43.3] |
| ≥6.9 to <7.4 (n = 16) | −295.7 ± 196.6 | −279.8 (−733.9, −54.1) | −55.5 [−66.7, −40.4] |
| ≥7.4 (*n* = 20) | −221.5 ± 260.5 | −213.9 (−573.9, 470.9) | −52.8 [−66.0, −34.7] |
| Duration of diabetes, year |  | | |
| <10 (*n* = 10) | −169.8 ± 312.2 | −97.5 (−733.9, 470.9) | −44.0 [−68.6, 0.0] |
| ≥10 to <20 (*n* = 26) | −283.8 ± 193.5 | −290.8 (−583.3, 195.1) | −56.0 [−65.0, −44.8] |
| ≥20 (*n* = 20) | −303.9 ± 185.3 | −283.3 (−786.0, 59.8) | −57.5 [−66.4, −46.3] |
| SGLT2 inhibitor use |  | | |
| Yes (*n* = 11) | −249.6 ± 302.5 | −278.5 (−733.9, 470.9) | −52.1 [−70.5, −22.2] |
| No (*n* = 45) | −275.8 ± 195.0 | −281.0 (−786.0, 195.1) | −55.2 [−62.2, −47.0] |
| DPP-4 inhibitor use |  | | |
| Yes (*n* = 34) | −294.8 ± 237.9 | −286.8 (−786.0, 470.9) | −56.9 [−65.2, −46.5] |
| No (*n* = 22) | −233.2 ± 179.3 | −245.8 (−514.1, 195.1) | −51.0 [−61.6, −37.5] |
| BMI, kg/m^2^ |  |  |  |
| <25 (*n* = 20) | −304.3 ± 223.7 | −328.5 (−786.0, 195.1) | −58.9 [−69.2, −45.1] |
| ≥25 (*n* = 36) | −251.9 ± 214.4 | −235.6 (−733.9, 470.9) | −52.1 [−60.6, −41.8] |
| Number of antihypertensive agents |  |  |  |
| Monotherapy (*n* = 14) | −271.9 ± 205.1 | −223.9 (−733.9, 51.5) | −54.5 [−67.8, −35.6] |
| Double therapy (*n* = 28) | −233.2 ± 244.5 | −275.6 (−786.0, 470.9) | −50.7 [−60.9, −37.8] |
| Triple therapy or more (*n* = 14) | −344.2 ± 156.4 | −386.6 (−583.3, −102.3) | −61.7 [−72.3, −47.1] |

*BMI* body mass index; *CI* confidence interval; *DPP-4* dipeptidyl peptidase 4; *eGFR* estimated glomerular filtration rate; *HbA1c* hemoglobin A1c; *SD* standard deviation; *SGLT2* sodium-glucose transport protein 2; *UACR* urinary albumin-to-creatinine ratio.
