# Supplementary material for: Efficacy and safety of esaxerenone (CS-3150) in Japanese patients with type 2 diabetes and macroalbuminuria: a multicenter, single-arm, open-label phase III study
Source: Clin Exp Nephrol. 2021 Jun 10;25(10):1070–8. doi: 10.1007/s10157-021-02075-y (PMC8421271; doi:10.1007/s10157-021-02075-y)

Electronic supplementary material

Efficacy and safety of esaxerenone (CS-3150) in Japanese patients with type 2 diabetes and macroalbuminuria: a multicenter, single-arm, open-label phase III study

Clinical and Experimental Nephrology

Sadayoshi Ito, Naoki Kashihara, Kenichi Shikata, Masaomi Nangaku, Takashi Wada, Yasuyuki Okuda, Tomoko Sawanobori

Corresponding author:

Sadayoshi Ito, MD, PhD

Division of Nephrology, Endocrinology and Vascular Medicine, Department of Medicine, Tohoku University School of Medicine, 2-1 Seiryo-machi, Aoba-ku, Sendai, Miyagi 980-8575, Japan

E-mail: db554@med.tohoku.ac.jp

Online Resource 1 Study design

ACE angiotensin converting enzyme; *ARB* angiotensin receptor blocker

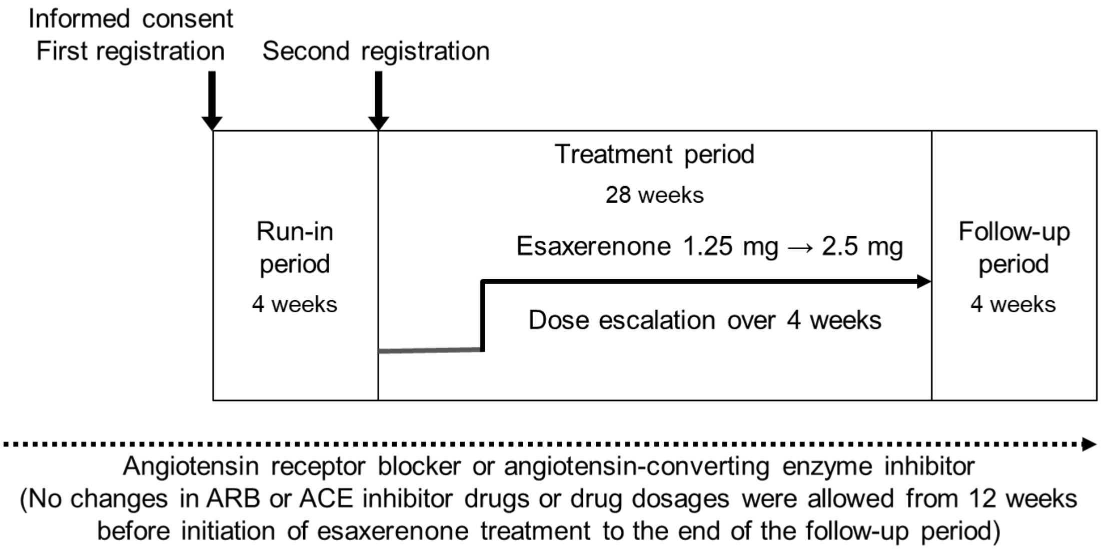

Supplement: Supplementary file 5 — Supplementary file5 (PDF 140 kb) [file 10157_2021_2075_MOESM5_ESM.pdf]
